# A Comprehensive Phenotypic Characterization of Biofilm-Producing Coagulase-Negative Staphylococci: Elucidating the Complexities of Antimicrobial Resistance and Susceptibility

**DOI:** 10.7759/cureus.79039

**Published:** 2025-02-15

**Authors:** Safa Modak, Priyanka Mane, Satish Patil

**Affiliations:** 1 Microbiology, Krishna Institute of Medical Sciences, Krishna Vishwa Vidyapeeth (Deemed to be University), Karad, IND

**Keywords:** antimicrobial sensitivity, biofilm producers, coagulase-negative staphylococcus, gram-positive cocci, nosocomial and opportunistic infections

## Abstract

Background

Coagulase-negative staphylococci (CoNS) have emerged as significant pathogens in nosocomial infections, particularly in bloodstream infections and individuals linked to embedded therapeutic devices. CoNS predominantly affects immunocompromised or debilitated patients. Additionally, individuals with medical indwelling devices, such as a catheter for the urinary tract, valves for the cardiovascular system, pacemakers, and prosthetic joints, should receive medical attention. As a result of the organism's evolving resistance to multiple antibiotics, managing CoNS infections is becoming increasingly challenging. The formation of biofilms is a key factor contributing to the pathogenicity and antimicrobial resistance of CoNS, complicating treatment efforts and the resolution of infections.

Aim

The aim of this study is to identify CoNS species, examine their biofilm production, and evaluate their resistance to antibiotics.

Materials and methods

A cross-sectional study was conducted on patients admitted to the Microbiology Department at Krishna Hospital, Karad. Clinical samples included the following: blood, pus, urine, sputum, endotracheal tube aspirate, high vaginal swab, and central venous catheter.

Results

The occurrence of coagulase-negative *Staphylococcus* across the range of clinical samples consisted of blood (61 isolates, 75.3%), pus (seven isolates, 8.6%), urine (six isolates, 7.4%), sputum (three isolates, 3.7%), endotracheal tubes (two isolates, 2.5%), and high vaginal swab and central venous catheter (one isolate (1.2%) each). The most often isolated species was *Staphylococcus haemolyticus* (35 isolates, 43.2%) followed by *Staphylococcus epidermidis* (22 isolates, 27.2%) and *Staphylococcus hominis* (12 isolates, 14.8%). We assessed the production of biofilms using Congo red agar, with 62 isolates (76.5%) demonstrating biofilm formation. Among these, *S. *haemolyticus** was the predominant species exhibiting biofilm production, with 29 isolates (46.8%) testing positive. This was followed by *S. *epidermidis** with 19 isolates (30.7%) and *S. *hominis** with nine isolates (14.5%).

Conclusion

The results of antibiotic susceptibility tests revealed multidrug resistance, with most isolates showing a high level of susceptibility to linezolid (84%) and complete resistance to penicillin. These findings highlight the clinical implications of limited treatment options and the need for alternative therapies, such as linezolid, in managing infections caused by coagulase-negative staphylococci.

## Introduction

*Staphylococcus* species, members of the staphylococcaceae family, are ubiquitous bacteria that play a dual role as both commensals and opportunistic pathogens in humans. They are Gram-positive, facultatively anaerobic, non-spore-forming, and immotile cocci capable of producing catalase. These bacteria are widely found on human skin and mucosal surfaces, where they generally remain harmless but can lead to infections in immunocompromised patients when conditions are favorable. Based on their ability to produce the coagulase enzyme, they are classified as coagulase-positive staphylococci (CoPS) or coagulase-negative staphylococci (CoNS) [1]. While *Staphylococcus lugdunensis* is categorized as CoNS, it exhibits clinical characteristics of both CoPS and CoNS, leading to its designation as an intermediate species [2].

CoNS primarily colonize human skin and mucous membranes as commensals. However, they are also recognized as opportunistic pathogens, particularly in healthcare settings, where they contribute significantly to healthcare-associated infections (HAIs) [3]. These organisms are of particular concern due to their increasing resistance to antimicrobial agents, which makes infection more challenging to manage [4]. Since they are commensals on human skin and mucosa, they were long believed to be non-pathogenic. In hospitals, they have lately been recognized as important nosocomial pathogens due to changes in medical practices and changes in host populations [5,6].

Although CoNS were historically considered non-pathogenic, they have emerged as major nosocomial pathogens due to advancements in medical interventions and increased susceptibility among hospitalized patients [7]. They are a leading cause of device-associated infections, particularly in immunocompromised individuals requiring long-term central venous access [8]. A key virulence factor of CoNS is their ability to produce biofilms, which are structured microbial communities embedded in a self-produced extracellular polymeric substance (EPS) [9]. Biofilm formation enhances bacterial survival by protecting against host immune responses and antimicrobial agents, leading to persistent and recurrent infections [10,11]. Notably, biofilm-producing strains of CoNS exhibit significantly higher resistance to antibiotics compared to their non-biofilm-producing counterparts [12].

CoNS, non-pathogenic native microbiota of the skin, nares, and other mucosal surfaces of humans, are opportunistic pathogens [13]. They infect immunocompromised and immunosuppressed patients and those with long-term hospitalizations and critical illnesses with nosocomial infections [14]. There are several types of CoNS, but the main origin of infections related to surgical wounds, catheters, osteomyelitis, peritonitis, and endophthalmitis is *Staphylococcus epidermidis* [15]. Several other members of the CoNS, including *Staphylococcus haemolyticus*, *Staphylococcus saprophyticus*, *Staphylococcus hominis*, *Staphylococcus warneri*, *Staphylococcus capitis*, *Staphylococcus simulans*, *Staphylococcus cohnii*, and *Staphylococcus xylosus*, are also acknowledged for their opportunistic role [14].

For an accurate diagnosis and effective management of CoNS infections, species-level identification, antimicrobial susceptibility testing, and biofilm detection are essential. It is also possible to develop targeted therapeutic strategies by understanding the genetic determinants of biofilm formation and resistance mechanisms. In order to control the spread of CoNS infections in healthcare settings, a multidisciplinary approach is essential that integrates the perspectives of microbiologists, clinicians, and epidemiologists [6].

## Materials and methods

Study location, type, and duration

A cross-sectional study on 81 CoNS isolates was conducted at Krishna Institute of Medical Sciences and Medical Research Centre, Karad, between March 2023 and March 2024. Ethical approval was obtained from the Institutional Ethical Committee (Approval No.: KIMSDU/IEC/02/2023) under protocol number 430/2022-2023.

Inclusion criteria

Various clinical samples that arrived at the microbiology laboratory were part of the study. Patients of all ages and genders were part of the research.

Exclusion criteria

Isolates from out-patients and repetitive isolates were excluded from the study if they were from the same patient.

Sample collection and processing

Bacterial cultures were retrieved from clinical specimens and identified using colony morphology, Gram staining, and biochemical assays. Initial screening included catalase, slide coagulase, and tube coagulase tests to differentiate CoNS from CoPS species.

For identifying CoNS at the species level, tests were conducted for carbohydrate fermentation (including xylose, sucrose, trehalose, mannitol, maltose, mannose, and lactose), ornithine decarboxylation, nitrate reduction, phosphatase production, urease production, and susceptibility to novobiocin and polymyxin B. Species identification was confirmed using standard microbiological methods [6,16].

All tests were conducted under aseptic conditions, and results were interpreted following established microbiological methodologies. Appropriate quality control strains were used throughout the process.

Detection of biofilm production

Biofilm production by all bacterial isolates was assessed using the Congo red agar (CRA) method, as described by Freeman et al. By examining colony morphology, it is possible to distinguish strains that produce biofilm from those that do not produce biofilm.

For CRA preparation, 37 g of brain heart infusion (BHI) broth, 50 g of sucrose, 0.8 g of Congo red dye, and 10 g of agar (all obtained from HiMedia, India) were dissolved in 1 L of distilled water. The medium was sterilized by autoclaving at 121°C for 15 minutes, followed by cooling to 55°C before pouring into sterile Petri dishes.

Each bacterial isolate was streaked onto the CRA medium and incubated aerobically at 37°C for 18-24 hours. Biofilm-producing strains appeared as black colonies with a dry, crystalline consistency, while non-biofilm producers formed red colonies with a smooth texture [3].

To ensure reliability, the assay was performed in duplicate, and known biofilm-producing and non-biofilm-producing *Staphylococcus* strains were used as positive and negative controls, respectively.

Antimicrobial susceptibility testing

Antimicrobial susceptibility testing was conducted using the Kirby-Bauer disk diffusion method, in accordance with the 2023 Clinical and Laboratory Standards Institute (CLSI) guidelines, to ensure standardized and reliable results. Bacterial suspensions were adjusted to a 0.5 McFarland turbidity standard and evenly inoculated onto Mueller-Hinton agar (MHA) plates using a sterile swab.

The following antimicrobial disks (HiMedia, India) were used: cefoxitin (30 µg), ciprofloxacin (5 µg), clindamycin (2 µg), erythromycin (15 µg), gentamicin (10 µg), linezolid (30 µg), penicillin (10 units), and tetracycline (30 µg) [17]. The plates were incubated at 37°C for 24 hours, after which the zone of inhibition was measured and interpreted according to CLSI breakpoints. Quality control was ensured using *Staphylococcus aureus* ATCC 25923 as a reference strain.

## Results

The study included 81 CoNS strains, collected in pure form from various clinical specimens. The distribution of these strains was as follows: blood 61 (75.3%), pus 7 (8.6%), urine 6 (7.4%), sputum 3 (3.7%), endotracheal tube 2 (2.5%), high vaginal swab 1 (1.2%), and central venous catheter 1 (1.2%). Statistical analysis revealed significant differences in the distribution of strains across specimen types (p < 0.05), with blood samples representing the largest proportion. Confidence intervals for the proportions were calculated to provide further statistical context (Table 1).

**Table 1:** CoNS surveillance in various clinical specimens CoNS: coagulase-negative *Staphylococcus*; ETT: endotracheal tube

Specimen	Overall count of CoNS	Percentage (%)
Blood	61	75.3
Pus	7	8.6
Urine	6	7.4
Sputum	3	3.7
ETT	2	2.5
High vaginal swab	1	1.2
Central venous catheter	1	1.2
Total	81	100

Blood specimens contained the highest count of CoNS. *S. haemolyticus* 35 (43.2%) was the predominant isolated species, followed by *S. epidermidis* 22 (27.2%) and *S. hominis* 12 (14.8%) (Table 2).

**Table 2:** Species surveillance of CoNS isolates CoNS: coagulase-negative staphylococci; *S. haemolyticus*: *Staphylococcus haemolyticus*; *S. epidermidis*: *Staphylococcus epidermidis*; *S. hominis*: *Staphylococcus hominis*; *S. capitis*: *Staphylococcus capitis*; *S. warneri*: *Staphylococcus warneri*; *S. lugdunensis*: *Staphylococcus lugdunensis*; *S. saprophyticus*: *Staphylococcus saprophyticus*; *S. xylosus*: *Staphylococcus xylosus*

Species	Total count of isolates	Percentage (%)
S. haemolyticus	35	43.2
S. epidermidis	22	27.2
S. hominis	12	14.8
S. capitis	3	3.7
S. warneri	4	4.9
S. lugdunensis	2	2.5
S. saprophyticus	1	1.2
S. xylosus	2	2.5
Total	81	100

Slime formation was observed in 62 (76.54%) of CoNS strains. Within these, *S. haemolyticus* ranked as the most prevalent species exhibiting biofilm production, with 29 isolates (46.8%) testing positive. This was followed by *S. epidermidis* with 19 isolates (30.7%) and *S. hominis* with nine isolates (14.5%) (Table 3).

**Table 3:** Species-based slime production *S. haemolyticus*: *Staphylococcus haemolyticus*; *S. epidermidis*: *Staphylococcus epidermidis*; *S. hominis*: *Staphylococcus hominis*; *S. capitis*: *Staphylococcus capitis*; *S. warneri*: *Staphylococcus warneri*; *S. lugdunensis*: *Staphylococcus lugdunensis*; *S. saprophyticus*: *Staphylococcus saprophyticus*; *S. xylosus*: *Staphylococcus xylosus*

Species	Slime producers	Percentage
S. haemolyticus	29	46.8
S. epidermidis	19	30.7
S. hominis	9	14.5
S. capitis	2	3.2
S. warneri	1	1.6
S. lugdunensis	1	1.6
S. saprophyticus	1	1.6
S. xylosus	0	0
Total	62	100

Antibiotic resistance testing is conducted using the Kirby-Bauer disk diffusion technique, adhering to CLSI guidelines. Resistance to multiple drugs was observed against commonly used antibiotics. All strains had resistance to penicillin. The highest susceptibility was observed for linezolid (68 strains, 83.95%). Cefoxitin serves as an indicator of methicillin resistance (Table 4).

**Table 4:** Antibiotic sensitivity pattern of coagulase-negative staphylococci

Antibiotics	Sensitive	Resistant	Sensitive (%)	Resistant (%)
Cefoxitin	17	64	21	79
Ciprofloxacin	19	62	23.5	76.5
Clindamycin	34	47	42	58
Erythromycin	11	70	13.6	86.4
Gentamycin	45	36	55.6	44.4
Linezolid	68	13	84	16
Penicillin	0	81	0	100

## Discussion

CoNS colonize human skin and mucous membranes. These opportunistic pathogens, primarily found in hospitals, have recently gained recognition as major hospital-acquired infections due to changes in medical practices and patient populations [3,6].

The increasing prevalence of drug-resistant microorganisms is driving a rise in HAIs, complicating treatment. CoNS infections are among the most common device-related infections, especially in immunocompromised and chronically ill patients needing long-term central venous access [4,7].

In healthcare settings, their protective slime layer helps them form colonies and spread, enhancing their ability to cause disease. Biofilm formation also reduces antibiotic effectiveness and protects microorganisms from the immune system, making them a significant public health threat [6,11]. Thus, testing for slime production is crucial for identifying the virulent traits of CoNS strains and should be routinely performed in diagnostic laboratories.

Our study included 81 samples of CoNS collected over a one-year period (March 2023-March 2024). We identified eight distinct species of CoNS among the isolates.

In our study, CoNS was found in various clinical samples, including blood (75.3%), pus (8.6%), urine (7.4%), sputum (3.7%), endotracheal tubes (2.5%), and high vaginal swabs and central venous catheters (1.2% each). The findings align with a study by Raina et al., where 45% of CoNS isolates were obtained from blood samples [18].

The higher frequency of CoNS isolation from blood samples in our study may be attributed to an increased number of blood samples tested for CoNS and the testing of more patients with risk factors (such as medical devices or immunocompromised conditions). However, contamination levels in bloodstream infections (BSIs) may also be elevated. In this study, CoPS isolates from blood samples were considered clinically significant and identified as pathogens only when they were recovered from paired blood samples from two peripheral veins. For patients with intravascular catheters, CoNS isolates from catheter tips were deemed significant and clinically relevant only if the same organism was found in the corresponding peripheral blood sample.

A range of species were identified in our study, including *S. haemolyticus* (43.2%), *S. epidermidis* (27.2%), and *S. hominis* (14.8%). The following species were found in much lower frequencies: *S. warneri* (4.9%), *S. capitis* (3.7%), *S. lugdunensis* (2.5%), *S. saprophyticus* (1.2%), and *S. xylosus* (2.5%).

Our research aligned with the findings of Raina et al., who identified *S. haemolyticus* (25%) as the most predominant species [18]. However, in their study, *S. warneri* (20%) and *S. epidermidis* (11.6%) were the second and third most common isolates, respectively.

In our study, the CRA method revealed that 46.8% of *S. haemolyticus* were biofilm producers, followed by 30.7% in *S. epidermidis* and 14.5% in *S. hominis*. These three species exhibited the highest biofilm production compared to *S. capitis*, *S. warneri*, *S. lugdunensis*, and *S. saprophyticus*, while *S. xylosus* did not produce any biofilm.

Antibiotic susceptibility testing was conducted using the Kirby-Bauer disk diffusion method according to CLSI guidelines. Multidrug resistance was observed against frequently used antibiotics, with all isolates showing resistance to penicillin. The highest sensitivity was observed to be linezolid (83.95%). Cefoxitin was used as a marker for methicillin resistance.

Limitations

This study was limited by a small sample size, which may not fully represent the diversity of CoNS species. Biofilm production was assessed only using the CRA method, which has limitations in sensitivity and specificity. The antimicrobial resistance testing focused on a limited range of antibiotics, and the study did not explore genetic mechanisms of biofilm formation and resistance.

Future studies should include a larger sample size from multiple healthcare settings and incorporate molecular techniques to better understand biofilm production and resistance. A broader range of antibiotics should be tested, and clinical studies should focus on treatment outcomes, especially in immunocompromised patients. Longitudinal studies tracking biofilm and resistance dynamics over time would provide valuable insights.

## Conclusions

CoNS have become prominent nosocomial pathogens, largely due to the increased use of intravascular devices and the rising number of immunocompromised patients in hospitals. The identification of CoNS and their antibiotic susceptibility profiles should be approached with care and diligence in clinical practice and epidemiological studies. The rising prevalence of multidrug-resistant CoNS, especially methicillin-resistant strains, poses significant concerns, as it not only restricts treatment options but also serves as a reservoir for antibiotic resistance genes.
